# Assessing measurement equivalence of the Danish and Dutch Four-Dimensional Symptom Questionnaire using differential item and test functioning analysis

**DOI:** 10.1177/1403494820942074

**Published:** 2020-07-27

**Authors:** Berend Terluin, Andreas Hoff, Lene Falgaard Eplov

**Affiliations:** 1 Department of General Practice and Elderly Care Medicine, Amsterdam Public Health Research Institute, Amsterdam UMC, Vrije Universiteit Amsterdam, The Netherlands; 2 Copenhagen Research Centre for Mental Health – CORE, Mental Health Centre Copenhagen, Copenhagen University Hospital, Denmark

**Keywords:** Distress, depression, anxiety, somatisation, unidimensionality, bi-factor model, measurement equivalence, measurement invariance, differential item functioning, differential test functioning

## Abstract

*Aims:* The Dutch Four-Dimensional Symptom Questionnaire (4DSQ) measures distress, depression, anxiety and somatisation, facilitating the distinction between stress-related problems and psychiatric disorder in primary and occupational health care. The aim of the study was to examine the measurement equivalence across the Danish and Dutch 4DSQ. *Methods:* Danish 4DSQ data were obtained from a cohort of Danish citizens on sick leave for mental-health problems. Dutch 4DSQ data were obtained from a cohort of Dutch employees on sick leave and a cohort of general practice attenders suspected of having mental-health problems. The study samples were matched on age and sex. The 4DSQ scales were assessed for essential unidimensionality using confirmatory factor analysis. Measurement equivalence of the 4DSQ across the groups was assessed using differential item and test functioning (DIF and DTF) analysis. *Results:* The study groups each consisted of 1363 people (63% female, *M*_age_=42 years). The 4DSQ scales proved essentially unidimensional. DIF was detected in 20 items. In terms of Cohen’s effect size, DIF was mostly small or moderate. In terms of effect size, the mean effect on the scale score (DTF) was negligible. Nevertheless, it is recommended to adjust some of the cut-off points for two Danish 4DSQ scales to retain the meaning of these cut-off points in Dutch respondents. *Conclusions:* **The Danish version of the 4DSQ measures the same constructs as the original Dutch questionnaire. Twenty items functioned differently in Danish respondents than in Dutch respondents, but this had only a small impact on the scale scores.**

## Introduction

Mental-health problems play an important role in sickness absence, in particular in long-term sickness absence [1,2]. The most prevalent problems are related to stress, depression, anxiety and medically unexplained physical symptoms (i.e. somatisation) [3,4]. These problems can be assessed using the four scales of the Dutch Four-Dimensional Symptom Questionnaire (4DSQ) [5]. The distress scale measures the kind of symptoms people experience when they feel ‘stressed’. The depression scale measures relatively specific symptoms of depressive disorder, such as anhedonia and negative cognitions, and indicates (moderate-severe) DSM-IV depressive disorder when the score is high [6]. The anxiety scale measures symptoms that are relatively specific to DSM-IV anxiety disorders, such as panic attacks, free floating anxiety and phobic fears. High anxiety scores indicate the presence of (severe) DSM-IV anxiety disorder, in particular panic disorder, agoraphobia, social phobia, post-traumatic stress disorder and obsessive-compulsive disorder [6,7]. The somatisation scale measures the kind of physical symptoms that are characteristic of somatoform disorder [8]. The 4DSQ is one of the few mental-health questionnaires having a distress scale alongside scales for depression and anxiety, thereby facilitating the distinction between stress-related problems (which are especially prevalent in primary and occupational health-care settings) and psychiatric disorder. Distress is the most general expression of mental problems, and as such it is associated with job stress, social difficulties and stressful life events [5]. Somatisation and distress are related to frequency and duration of sickness absence [9]. Somatisation is also related to health-care utilisation [10]. In The Netherlands, the 4DSQ is widely used in primary care and occupational health care to detect mental-health problems and, above all, to help make mental problems a topic for discussion in the doctor’s surgery. The 4DSQ is incorporated in several Dutch professional guidelines for primary and occupational health care. The 4DSQ has successfully been translated into various languages, including English, French, German, Polish, Turkish and Arabic [11–15].

The Integreret Behandlings- og Beskæftigelses-Indsats til Sygemeldte (IBBIS) study offered integrated mental-health care and vocational rehabilitation to individuals on sick leave due to mental-health problems. The study consisted of two randomised controlled trials (RCTs): one for stress-related problems and one for depression and anxiety [16,17]. Since, unlike most mental-health questionnaires, the 4DSQ covers both domains, this questionnaire was chosen as the main measure of mental-health problems in both RCTs. Using the same questionnaire across both trials also facilitates the cross-study comparison of outcomes. We developed a Danish 4DSQ version using forward and backward translation. However, a translated questionnaire cannot be assumed to possess the same measurement properties of the original questionnaire, even after careful translation [18]. Translated items may differ slightly from the original ones in meaning or severity, potentially making Danish and Dutch 4DSQ scores incomparable. Therefore, in this paper, we assessed the measurement equivalence across the Danish and Dutch 4DSQ.

## Methods

### Study population

The study population was selected from three source populations derived from one Danish study and two Dutch studies. The Danish source population consisted of citizens on sick leave due to mental-health problems, who were assessed for the IBBIS study in Danish job centres in four municipalities in Denmark [16,17] and who had completed the Danish 4DSQ at baseline. The IBBIS study was approved by regional ethics committees of the capital region, and participants gave informed consent [16,17]. The first Dutch source population consisted of employees on sick leave for any reason [4]. In this group, a mental disorder was diagnosed in 43% by the occupational physician. The study was approved by the Medical Ethics Committee of the University Medical Centre in Groningen, and participants provided informed consent [4]. The second Dutch source population consisted of general practice attenders suspected by their general practitioner of having mental-health problems [19]. In the latter population, the data were collected during routine primary care for which ethical approval and informed consent were not applicable.

The Dutch groups were merged. Persons with item scores missing for more than half of the items of any of the 4DSQ scales were excluded. The study population to be used in the present study was selected from the Danish and Dutch source populations by matching for sex and age (10-year groups) in such a way that each sex/age stratum contained equal numbers of Danish and Dutch people, while the size of each stratum was maximised given the available people in the source populations. Remaining missing item scores were imputed using the response function method [20], a method based on non-parametric item response theory (IRT) that takes both differences between people and differences between items into account [21].

### Measurement

The 4DSQ is a 50-item self-report questionnaire consisting of four scales measuring distress (16 items), depression (6 items), anxiety (12 items) and somatisation (16 items) [5]. The 4DSQ items are scored on a five-point scale, but in order to neutralise exaggerating response tendencies, the scores are recoded into a three-point scale (0=‘no’, 1=‘sometimes’, 2=‘regularly’, ‘often’ and ‘very often or constantly’). For each scale, two cut-off scores are employed to distinguish between ‘low’, ‘moderate’ and ‘severe’ scores. The validity of the 4DSQ was evaluated by comparing to other questionnaires and clinical diagnoses [5,6]. The reliability of the 4DSQ scales proved to be good, with Cronbach’s alpha values well above 0.8 and McDonald’s omega values well above 0.9 [5,22].

One member of the IBBIS research team developed an English-to-Danish translation of the 4DSQ, which was subsequently independently back-translated by an external translator. The final version was based on discussion between the translators.

### Statistical analysis

#### Measurement equivalence

Measurement equivalence across two (language) versions of a scale means that the versions measure the same construct in the same way. Scales measure unobservable constructs (also called latent traits), such as depression, using items (i.e. questions) that elicit responses that are deemed indicative of the trait of interest. Regarding their ability to convey information about the trait, items may vary in their ‘severity’ and ‘discrimination’ characteristics. Severity refers to the level of the trait about which an item is particularly informative. Discrimination refers to how well an item is able to separate respondents who are relatively high on the trait from respondents who are relatively low. Measurement equivalence across two versions of a scale implies that the corresponding items of the scale versions possess similar severity and discrimination characteristics. This can be examined using differential item functioning (DIF) analysis [23]. We chose to use DIF analysis within the framework of IRT because IRT directly models the relationship between item responses and the underlying trait, estimating the item characteristics as parameters of the statistical model [24].

#### Dimensionality

The application of IRT requires a scale to be ‘essentially unidimensional’ [25]. This means that the item responses are predominantly driven by a single large general factor, and that additional smaller factors do not impact the scale scores too much. We assessed the dimensionality of each of the 4DSQ scales in each language group using bi-factor analysis within a structural equation modelling framework [26]. The item responses were treated as ordered categories. After fitting a one-factor measurement model (largely identifying the general factor), residual correlations were used to identify smaller ‘specific’ factors, which were subsequently added to a bi-factor model until adequate model fit was achieved. Factor loadings of specific factors defined by only two items were constrained to be equal to make the model estimable. The following scaled fit indices were taken as indicative of adequate fit: comparative fit index >0.95, Tucker–Lewis index >0.95, root mean square error of approximation <0.06 and standardised root mean squared residual <0.08 [27]. The following bi-factor statistics were taken as indicative of essential unidimensionality: the proportion of uncontaminated correlations >0.8, the explained common variance >0.6 or omega-hierarchical >0.8 [28].

#### DIF

DIF analysis implies testing the equality of item parameters (difficulty and discrimination) across two groups. We used an IRT approach involving three stages to identify appropriate ‘anchor’ items to link the groups on the same latent trait scale [29,30]. First, a unidimensional multi-group graded response model (GRM) was fitted to the scale, constraining the item parameters across the groups while freely estimating the latent mean and variance of the focal group relative to the reference group. This first step actually assumed that all items together measure approximately the same construct in about the same way. Second, a new GRM was fitted using the estimated latent mean and variance to link the groups on a common latent scale while freely estimating the parameters of all items. The Wald test was then used to test differences in item parameters across the groups and to identify DIF-free items (*p*>0.05). Third, the items without DIF were then used as anchor items in a third GRM constraining the item parameters of the anchor items while freely estimating the parameters of the other items and the latent mean and variance. The Wald test was used again to test for DIF in the non-anchor items. Items with Bonferroni corrected *p*-values <0.001 and unsigned item difference in the sample (UIDS) values >0.1 (see below) were identified as DIF items. To assess the magnitude of DIF, a final GRM was then fitted in which the parameters of the DIF items, and the latent mean and variance were freely estimated while the parameters of the non-DIF items were constrained. The magnitude of DIF was then expressed as effect sizes based on expected item scores calculated twice based on either the item parameters in the reference group or the item parameters in the focal group [31]. The signed item difference in the sample (SIDS) represents the mean difference in expected item scores across the groups. The UIDS represents the mean of the absolute difference in expected item scores across the groups. Unlike the SIDS, the UIDS does not allow for cancellation of differences across respondents. The SIDS and UIDS are expressed in the metric of the scale score. In addition, we calculated the expected score standardised difference (ESSD), which is the Cohen’s *d* version of the SIDS. Absolute ESSD values <0.2 can be interpreted as negligible DIF, 0.2–0.5 as small DIF, 0.5–0.8 as moderate DIF and >0.8 as large DIF.

#### Differential test functioning

DIF causes higher item scores in one group compared to the other group without there being a difference in the true level of the underlying trait. However, DIF does not need to have a large impact on the scale score, that is, differential test functioning (DTF). We assessed DTF by calculating a number of scale-level effect sizes [31]. The signed test difference in the sample (STDS) is the sum of all SIDSs across the items of a scale. The unsigned test difference in the sample (UTDS) is the sum of all UIDSs across the items of a scale. The UTDS allows no cancellation across items or persons. The unsigned expected test score difference in the sample (UETSDS) is the average of absolute values of the expected test score differences in persons. As the UETSDS allows for cancellation across items but not across persons, the UETSDS reflects the true effect of DIF on scale scores. The expected test score standardised difference (ETSSD) is the Cohen’s *d* version of the STDS.

### Software

We used IBM SPSS Statistics for Windows v22 (IBM Corp., Armonk, NY) to prepare the data and impute missing item responses. We used ‘lavaan’ v06-2 for dimensionality analysis [32] and ‘mirt’ v1.26.3 for DIF and DTF analysis [33]. The software packages ‘lavaan’ and ‘mirt’ were used within R v3.5.1 (The R Foundation for Statistical Computing, Vienna, Austria).

## Results

### Descriptives

In the Danish source population, 2058 respondents were available, and in the Dutch source population, 1493 (497 sick-listed employees and 996 general practice attenders) were available. After matched selection, 1363 respondents remained in each language group. Percentages of missing item scores needing imputation were 0.16% in the Danish group and 0.63% in the Dutch group. Table I presents the study groups with respect to sex, age and 4DSQ scores.

**Table I.** Participant characteristics by language group.

Characteristics	Danish	Dutch
*N*	1363	1363
Sex (% female)	63.1	63.1
Age (years), *M* (*SD*)	42.0 (10.8)	41.8 (10.8)
4DSQ distress (range 0–32), *M* (*SD*)	19.7 (7.6)	18.5 (9.7)
4DSQ depression (range 0–12), *M* (*SD*)	3.4 (3.4)	3.7 (4.0)
4DSQ anxiety (range 0–24), *M* (*SD*)	6.5 (6.1)	6.3 (6.5)
4DSQ somatisation (range 0–32), *M* (*SD*)	12.4 (7.1)	14.0 (7.8)

### Dimensionality

The bi-factor models achieved adequate fit (see Supplemental Table SI; factor loadings are presented in Supplemental Table SII). The dimensionality statistics indicated that the 4DSQ scales were essentially unidimensional in both groups (see Supplemental Table SIII).

### Differential item functioning

**Figure 1.**
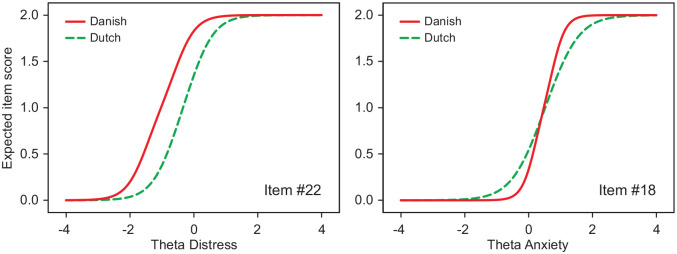
Examples of differential item functioning: expected item scores in relation to the underlying trait for two items for Danish and Dutch respondents.

DIF was found in 20 items across three scales (Table II; see Supplemental Table SIV for the item parameters). The depression scale was free of DIF. The SIDS values indicate that eight items were less severe for Danish respondents (positive SIDS values), and 12 items were more severe (negative SIDS values). For instance, the SIDS value for item #22 indicates that Danish respondents scored on average 0.394 point higher on item #22 than Dutch respondents with comparable levels of distress would do. To illustrate DIF, Figure 1 displays the expected item score in relation to the latent trait for items #22 and #18. Danish respondents started scoring on item #22 at much lower levels of distress than Dutch respondents did. For the Danish respondents, item #22 corresponded to a less severe level of distress than for the Dutch. Most DIF items, such as item #22, showed a difference in item severity across the groups. Only one item (#18) showed DIF due to a difference in item discrimination. In Figure 1, this is apparent by a difference in the slopes of the curves. For the Danish respondents, item #18 was slightly more discriminative than for the Dutch. This led to Danish respondents scoring slightly higher on item #18 than the Dutch in the higher range of the trait, but scoring slightly lower in the lower range. This is also apparent in the difference between the SIDS and the UIDS for item #18. The UIDS indicates that Danish respondents on average would score 0.167 point higher or lower on item #18 than Dutch respondents with comparable levels of anxiety if the DIF would have operated in the same direction across the range of the scale. The ESSD values in Table II indicate that in terms of effect size the DIF was large in four items (|ESSD| >0.8) and moderate in six items (|ESSD| 0.5–0.8).

**Table II.** Items with differential item functioning (DIF): effect sizes.

Scale	Item	Short item description (English)	SIDS	UIDS	ESSD
Distress	#22	Lack of energy	0.394	0.394	1.015
	#25	Feeling tense	−0.195	0.195	−0.523
	#29	Just can’t do anything anymore	−0.228	0.228	−0.374
	#32	Can’t cope anymore	−0.326	0.327	−0.518
	#36	Can’t face it anymore	−0.238	0.238	−0.374
	#48	Have to put aside thoughts of upsetting events	0.111	0.118	0.270
Anxiety	#18	Sudden fright	−0.024	0.167	−0.040
	#27	Frightened	−0.436	0.436	−0.711
	#42	Specific phobia	−0.177	0.177	−0.370
	#49	Avoid places that frightened you	0.143	0.143	0.330
	#50	Have to repeat some actions	−0.227	0.227	−0.801
Somatisation	#1	Dizziness or light-headed	−0.402	0.402	−1.022
	#2	Painful muscles	−0.460	0.460	−1.232
	#5	Back pain	−0.120	0.131	−0.342
	#6	Excessive sweating	−0.120	0.122	−0.320
	#7	Palpitations	0.268	0.268	0.603
	#9	Bloated feeling in the abdomen	0.248	0.248	0.543
	#12	Nausea or upset stomach	0.188	0.188	0.395
	#13	Pain in the abdomen or stomach	0.169	0.171	0.371
	#14	Tingling in the fingers	−0.161	0.161	−0.526

SIDS: signed item difference in the sample; UIDS: unsigned item difference in the sample; ESSD: expected score standardised difference.

### DTF

**Figure 2.**
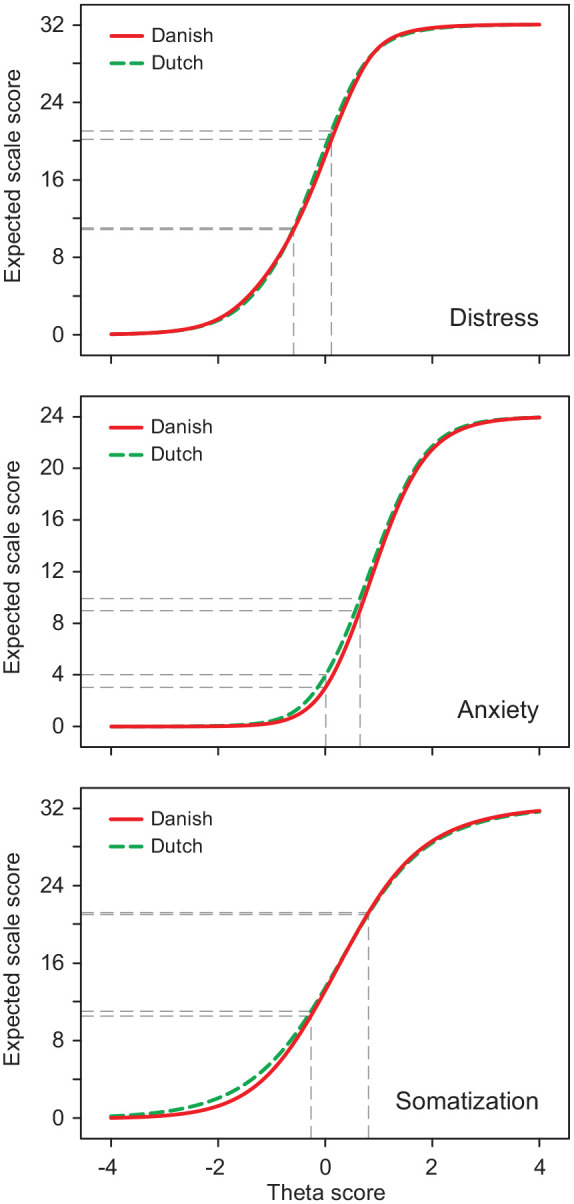
Differential test functioning: test characteristic curves, that is, the expected test scores in relation to the underlying trait for Danish and Dutch respondents. Conventional cut-off scores are indicated by dashed lines.

The impact of DIF on the scale level was negligible in terms of effect size (Table III). Because most of the DIF items in the anxiety scale were more severe for the Danish respondents than for the Dutch, the Danish respondents scored on average 0.721 point lower on the anxiety scale than the Dutch while having comparable levels of the anxiety trait. Figure 2 displays the test characteristic curves for the DIF-containing scales, that is, the expected test scores as a function of the latent trait by group. This shows that the relationship between the 4DSQ scale scores with the underlying traits were very similar in Danish and Dutch people, indicating that the Danish 4DSQ scales measured the 4DSQ dimensions as well as the Dutch 4DSQ. However, if we zoom in on the conventional (Dutch) cut-off points (in Figure 2 indicated by dashed lines), we can see a small difference between groups. The Dutch cut-off for moderate anxiety (i.e. 4) corresponded to a certain level of anxiety (θ=0), which in turn corresponded to an anxiety score of ~3 in Danish respondents. Thus, Danish respondents at the threshold of moderate anxiety scored around one point lower on the 4DSQ anxiety scale than Dutch respondents having the same level of anxiety. This difference between Danish and Dutch respondents could also be observed at the cut-offs for severe anxiety (9 vs. 10) and severe distress (20 vs. 21).

**Table III.** Differential test functioning (DTF): effect sizes.

Scale	STDS	UTDS	UETSDS	ETSSD
Distress	−0.482	1.500	0.545	−0.069
Anxiety	−0.721	1.151	0.721	−0.128
Somatisation	−0.390	2.152	0.437	−0.063

STDS: signed test difference in the sample; UTDS: unsigned test difference in the sample; UETSDS: unsigned expected test score difference in the sample; ETSSD: expected test score standardised difference.

## Discussion

### Main findings and implications

This study examined measurement equivalence across the Danish translation of the 4DSQ and its original Dutch version. That is, we investigated whether the Danish 4DSQ measures the same constructs as the original Dutch 4DSQ, whether Danish 4DSQ scores can be interpreted the same way as Dutch 4DSQ scores and whether specific (Dutch) cut-off scores can be used in Danish populations. We found that 20 items showed evidence of differential functioning, some of them even to a moderate or large extent in terms of effect size. However, much of the item-level DIF appeared to be cancelled out at the scale level. For instance, the effect of five somatisation items that were more severe for Danish respondents was largely counteracted by the effect of four somatisation items that were less severe.

For most DIF items, after carefully comparing Danish and Dutch item content, we were unable to come up with an explanation, except in one case: item #22. The Danish translation followed the English translation: lack of energy (in Danish: ‘mangel på energi’). However, the Dutch item refers to listlessness (in Dutch: ‘lusteloosheid’). Apparently, ‘mangel på energi’ is a much less severe symptom of distress for Danish people than ‘lusteloosheid’ is for the Dutch. Note, however, that the DIF in item #22 represented a blessing in disguise. As four other distress symptoms turned out to be more severe for Danish people, the DIF in item #22 was more than welcome to counteract the effect of DIF on the distress score. For this reason, we do not recommend fixing the DIF in item #22.

Our findings indicate that the 4DSQ scales measure the same constructs across Danish and Dutch people and that Danish 4DSQ scores for depression and somatisation can be interpreted exactly in the same way as Dutch 4DSQ scores. However, Danish respondents tended to score somewhat lower on the anxiety and distress scales than Dutch respondents would do, given their true levels of the constructs. This is particularly true for the cut-off points used for moderate and severe anxiety and for the cut-off point for severe distress. Therefore, it is worth considering reducing these cut-off points by one point for Danish respondents in order to retain the same meaning of the cut-off points across the groups. Given the standard error of measurement being 1.5 for distress and 1.4 for anxiety, a one-point difference does not really matter for the interpretation of individual 4DSQ scores. However, it may be relevant to take into account when evaluating or comparing group statistics (e.g. mean scores or percentages exceeding a cut-off point).

## Conclusions

The Danish version of the 4DSQ measures the same constructs as the original Dutch questionnaire. Twenty items functioned differently in Danish respondents than in Dutch respondents, but this had only a small impact on the scale scores.

## Supplemental Material

SJP942074_Supplemental_Table_1 – Supplemental material for Assessing measurement equivalence of the Danish and Dutch Four-Dimensional Symptom Questionnaire using differential item and test functioning analysis Click here for additional data file. Supplemental material, SJP942074_Supplemental_Table_1 for Assessing measurement equivalence of the Danish and Dutch Four-Dimensional Symptom Questionnaire using differential item and test functioning analysis by Berend Terluin, Andreas Hoff and Lene Falgaard Eplov in Scandinavian Journal of Public Health

SJP942074_Supplemental_Table_2 – Supplemental material for Assessing measurement equivalence of the Danish and Dutch Four-Dimensional Symptom Questionnaire using differential item and test functioning analysis Click here for additional data file. Supplemental material, SJP942074_Supplemental_Table_2 for Assessing measurement equivalence of the Danish and Dutch Four-Dimensional Symptom Questionnaire using differential item and test functioning analysis by Berend Terluin, Andreas Hoff and Lene Falgaard Eplov in Scandinavian Journal of Public Health

SJP942074_Supplemental_Table_3 – Supplemental material for Assessing measurement equivalence of the Danish and Dutch Four-Dimensional Symptom Questionnaire using differential item and test functioning analysis Click here for additional data file. Supplemental material, SJP942074_Supplemental_Table_3 for Assessing measurement equivalence of the Danish and Dutch Four-Dimensional Symptom Questionnaire using differential item and test functioning analysis by Berend Terluin, Andreas Hoff and Lene Falgaard Eplov in Scandinavian Journal of Public Health

SJP942074_Supplemental_Table_4 – Supplemental material for Assessing measurement equivalence of the Danish and Dutch Four-Dimensional Symptom Questionnaire using differential item and test functioning analysis Click here for additional data file. Supplemental material, SJP942074_Supplemental_Table_4 for Assessing measurement equivalence of the Danish and Dutch Four-Dimensional Symptom Questionnaire using differential item and test functioning analysis by Berend Terluin, Andreas Hoff and Lene Falgaard Eplov in Scandinavian Journal of Public Health
