# Supplementary material for: Assessing measurement equivalence of the Danish and Dutch Four-Dimensional Symptom Questionnaire using differential item and test functioning analysis
Source: Scand J Public Health. 2020 Jul 27;49(4):479–86. doi: 10.1177/1403494820942074 (PMC8135249; doi:10.1177/1403494820942074)
Supplement: SJP942074_Supplemental_Table_1 – Supplemental material for Assessing measurement equivalence of the Danish and Dutch Four-Dimensional Symptom Questionnaire using differential item and test functioning analysis [file SJP942074_Supplemental_Table_1.pdf]

Supplementary Table 1. Scaled fit indices of the bifactor models, by 4DSQ scale and language group

Scale/group	χ^2	df	p	CFI	TLI	RMSEA	90% CI	SRMR
Distress								
Danish	296.2	59.6	0.000	0.978	0.992	0.054	0.047, 0.061	0.037
Dutch	302.0	55.3	0.000	0.985	0.996	0.057	0.050, 0.065	0.028
Depression								
Danish	6.26	3.9	0.175	1.000	1.000	0.021	0.000, 0.070	0.008
Dutch	14.04	4.0	0.007	1.000	0.999	0.043	0.005, 0.086	0.009
Anxiety								
Danish	172.5	38.9	0.000	0.982	0.993	0.050	0.040, 0.060	0.034
Dutch	166.1	39.3	0.000	0.988	0.996	0.049	0.039, 0.059	0.033
Somatization								
Danish	232.8	63.2	0.000	0.973	0.987	0.044	0.038, 0.051	0.037
Dutch	208.7	63.3	0.000	0.980	0.991	0.041	0.034, 0.048	0.037

CFI = comparative fit index, TLI = Tucker-Lewis index, RMSEA = root mean square error or approximation, 90% CI = 90% confidence interval of the RMSEA, SRMR = standardized root mean squared residual
