# Supplementary material for: Assessing measurement equivalence of the Danish and Dutch Four-Dimensional Symptom Questionnaire using differential item and test functioning analysis
Source: Scand J Public Health. 2020 Jul 27;49(4):479–86. doi: 10.1177/1403494820942074 (PMC8135249; doi:10.1177/1403494820942074)
Supplement: SJP942074_Supplemental_Table_2 – Supplemental material for Assessing measurement equivalence of the Danish and Dutch Four-Dimensional Symptom Questionnaire using differential item and test functioning analysis [file SJP942074_Supplemental_Table_2.pdf]

**Supplementary Table 2. Factor loadings of the unidimensional and bifactor models**

Explanation:

The tables present standardized factor loadings of the unidimensional models and the bifactor models per 4DSQ scale per language group. In addition, the following bifactor statistics are presented: omega-total (omega-t), omega-hierarchical (omega-h), omega-subgroup (omega-s) and explained common variance (ECV). The omega-coefficients are based on a partitioning of the test-level (scale score) variance. Omega-t represents the test-level variance accounted for by all factors, divided by the total test-level variance (including error variance). As a reliability estimate, omega-t is comparable with Cronbach's alpha. Omega-h represents the test-level variance accounted for by the general factor, divided by the total test-level variance. Omega-h is a measure of the general factor saturation, i.e., as a measure of the reliability of the general factor. Omega-s represents the test-level variance of a subset of items making up a specific factor (i.e., subscale), accounted for by the specific factor, divided by the total variance of the subset of items. Omega-s thus is the proportion of test-level variance that the subscale items add over and beyond the variance accounted for by the general factor. The ECV represents the proportion of the total reliable item-level variance that is accounted by the respective factors.

The bifactor model of distress in Danish respondents (see below) show that 70.6% of the total reliable item-level variance was accounted for by the general factor (ECV). The specific factors accounted for much less item-level variance: 8.7%, 7.9%, 2.9% and 12.7% respectively. Omega-t of the distress scale (16 items) indicates that the proportion reliable test-level variance (accounted for by 4 factors) was 0.961. The general factor alone accounted for 88% of the total test-level variance (omega-h). If we would use a subscale of items #20 and #39 (making up factor F1), this subscale would account for a reliable proportion of test-level variance of 0.534 (omega-s) over and beyond the general factor. The omega-s values indicate that F1 and F2 do contribute some reliable variance (i.e., about half of the total subscale variance) to what is already captured by the general factor, but this cannot be said of factor F3 (i.e., only 27% of the 6 item subscale variance can be accounted for by these items; the rest is accounted for by the general factor and error).

| **Distress Danish** | | **Unidim. model** | **Bifactor model** | | | | |
|---|---|---|---|---|---|---|---|
| | **Item** | | **Gen** | **F1** | **F2** | **F3** | **F4** |
| | DSQ17 | 0.777 | 0.764 | | | 0.232 | |
| | DSQ19 | 0.735 | 0.788 | | | | |
| | DSQ20 | 0.607 | 0.550 | 0.683 | | | |
| | DSQ22 | 0.728 | 0.782 | | | | |
| | DSQ25 | 0.665 | 0.717 | | | | |
| | DSQ26 | 0.599 | 0.647 | | | | |
| | DSQ29 | 0.827 | 0.694 | | | 0.507 | |
| | DSQ31 | 0.768 | 0.686 | | | 0.400 | |
| | DSQ32 | 0.913 | 0.711 | | | 0.638 | |
| | DSQ36 | 0.874 | 0.683 | | | 0.600 | |
| | DSQ37 | 0.805 | 0.748 | | | 0.351 | |
| | DSQ38 | 0.725 | 0.787 | | | | |
| | DSQ39 | 0.567 | 0.513 | 0.683 | | | |
| | DSQ41 | 0.585 | 0.632 | | | | |
| | DSQ47 | 0.665 | 0.559 | | 0.651 | | |
| | DSQ48 | 0.729 | 0.653 | | 0.651 | | |
| Omega-t | | | 0.961 | | | | |
| Omega-h | | | 0.880 | | | | |
| Omega-s | | | | 0.534 | 0.474 | 0.272 | |
| ECV | | | 0.706 | 0.087 | 0.079 | 0.127 | |

| **Distress Dutch** | | **Unidim. model** | **Bifactor model** | | | | |
|---|---|---|---|---|---|---|---|
| | **Item** | | **Gen** | **F1** | **F2** | **F3** | **F4** |
| | DSQ17 | 0.827 | 0.870 | | | | |
| | DSQ19 | 0.837 | 0.870 | | | | |
| | DSQ20 | 0.719 | 0.685 | 0.614 | | | |
| | DSQ22 | 0.824 | 0.815 | | | 0.193 | |
| | DSQ25 | 0.866 | 0.901 | | | | |
| | DSQ26 | 0.791 | 0.827 | | | | |
| | DSQ29 | 0.882 | 0.812 | | | 0.388 | |
| | DSQ31 | 0.865 | 0.785 | | | 0.417 | |
| | DSQ32 | 0.914 | 0.814 | | | 0.461 | |
| | DSQ36 | 0.929 | 0.830 | | | 0.456 | |
| | DSQ37 | 0.918 | 0.833 | | | 0.425 | |
| | DSQ38 | 0.784 | 0.772 | | | 0.208 | |
| | DSQ39 | 0.649 | 0.604 | 0.614 | | | |
| | DSQ41 | 0.761 | 0.800 | | | | |
| | DSQ47 | 0.771 | 0.616 | | 0.678 | | |
| | DSQ48 | 0.806 | 0.674 | | 0.678 | | |
| Omega-t | | | 0.980 | | | | |
| Omega-h | | | 0.922 | | | | |
| Omega-s | | | | 0.421 | 0.491 | 0.162 | |
| ECV | | | 0.787 | 0.060 | 0.073 | 0.080 | |

| Depression Danish | | Unidim. model | Bifactor model | | |
|---|---|---|---|---|---|
| | **Item** | | **Gen** | **F1** | |
| | DSQ28 | 0.856 | 0.909 | | |
| | DSQ30 | 0.929 | 0.897 | | 0.319 |
| | DSQ33 | 0.970 | 0.822 | | 0.518 |
| | DSQ34 | 0.812 | 0.805 | 0.300 | |
| | DSQ35 | 0.827 | 0.831 | 0.300 | |
| | DSQ46 | 0.948 | 0.778 | | 0.578 |
| Omega-t | | | 0.970 | | |
| Omega-h | | | 0.887 | | |
| Omega-s | | | | 0.102 | 0.237 |
| ECV | | | 0.828 | 0.035 | 0.137 |

| Depression Dutch | | Unidim. model | Bifactor model | | |
|---|---|---|---|---|---|
| | **Item** | | **Gen** | **F1** | |
| | DSQ28 | 0.917 | 0.908 | 0.151 | |
| | DSQ30 | 0.947 | 0.977 | | |
| | DSQ33 | 0.980 | 0.951 | | 0.277 |
| | DSQ34 | 0.881 | 0.834 | 0.424 | |
| | DSQ35 | 0.885 | 0.842 | 0.341 | |
| | DSQ46 | 0.953 | 0.918 | | 0.277 |
| Omega-t | | | 0.981 | | |
| Omega-h | | | 0.944 | | |
| Omega-s | | | | 0.105 | 0.079 |
| ECV | | | 0.912 | 0.059 | 0.028 |

| Anxiety Danish | | Unidim. model | Bifactor model | | |
|---|---|---|---|---|---|
| | **Item** | | **Gen** | **F1** | **F2** |
| | DSQ18 | 0.889 | 0.791 | 0.428 | |
| | DSQ21 | 0.830 | 0.674 | 0.630 | |
| | DSQ23 | 0.688 | 0.717 | | |
| | DSQ24 | 0.823 | 0.772 | 0.300 | |
| | DSQ27 | 0.838 | 0.743 | 0.423 | |
| | DSQ40 | 0.782 | 0.812 | | |
| | DSQ42 | 0.729 | 0.761 | | |
| | DSQ43 | 0.804 | 0.832 | | |
| | DSQ44 | 0.695 | 0.724 | | |
| | DSQ45 | 0.789 | 0.761 | 0.221 | |
| | DSQ49 | 0.755 | 0.784 | | |
| | DSQ50 | 0.583 | 0.604 | | |
| Omega-t | | | 0.951 | | |
| Omega-h | | | 0.906 | | |
| Omega-s | | | | 0.207 | |
| ECV | | | 0.883 | 0.117 | |

| Anxiety Dutch | | Unidim. model | Bifactor model | | |
|---|---|---|---|---|---|
| | **Item** | | **Gen** | **F1** | **F2** |
| | DSQ18 | 0.783 | 0.807 | | |
| | DSQ21 | 0.897 | 0.805 | 0.444 | |
| | DSQ23 | 0.771 | 0.790 | | |
| | DSQ24 | 0.896 | 0.836 | 0.353 | |
| | DSQ27 | 0.935 | 0.843 | 0.458 | |
| | DSQ40 | 0.848 | 0.864 | | |
| | DSQ42 | 0.822 | 0.845 | | |
| | DSQ43 | 0.826 | 0.839 | | |
| | DSQ44 | 0.754 | 0.771 | | |
| | DSQ45 | 0.745 | 0.723 | 0.199 | |
| | DSQ49 | 0.812 | 0.828 | | |
| | DSQ50 | 0.649 | 0.661 | | |
| Omega-t | | | 0.962 | | |
| Omega-h | | | 0.941 | | |
| Omega-s | | | | 0.160 | |
| ECV | | | 0.931 | 0.069 | |

| **Somatization Danish** | | **Unidim. model** | **Bifactor model** | | | | |
|---|---|---|---|---|---|---|---|
| | **Item** | | **Gen** | **F1** | **F2** | **F3** | **F4** |
| | DSQ01 | 0.577 | 0.612 | | | | 0.543 |
| | DSQ02 | 0.626 | 0.599 | | | 0.386 | |
| | DSQ03 | 0.538 | 0.542 | | | | 0.543 |
| | DSQ04 | 0.590 | 0.488 | | | 0.730 | |
| | DSQ05 | 0.589 | 0.509 | | | 0.543 | |
| | DSQ06 | 0.558 | 0.605 | | | | |
| | DSQ07 | 0.650 | 0.594 | 0.383 | | | |
| | DSQ08 | 0.551 | 0.553 | | | 0.214 | |
| | DSQ09 | 0.676 | 0.612 | | 0.458 | | |
| | DSQ10 | 0.667 | 0.728 | | | | |
| | DSQ11 | 0.697 | 0.663 | 0.328 | | | |
| | DSQ12 | 0.715 | 0.654 | | 0.462 | | |
| | DSQ13 | 0.737 | 0.648 | | 0.616 | | |
| | DSQ14 | 0.553 | 0.599 | | | | |
| | DSQ15 | 0.783 | 0.618 | 0.723 | | | |
| | DSQ16 | 0.784 | 0.638 | 0.548 | | | |
| Omega-t | | | 0.940 | | | | |
| Omega-h | | | 0.841 | | | | |
| Omega-s | | | | 0.339 | 0.337 | 0.353 | 0.362 |
| ECV | | | 0.628 | 0.115 | 0.085 | 0.109 | 0.063 |

| **Somatization Dutch** | | **Unidim. model** | **Bifactor model** | | | | |
|---|---|---|---|---|---|---|---|
| | **Item** | | **Gen** | **F1** | **F2** | **F3** | **F4** |
| | DSQ01 | 0.690 | 0.742 | | | | |
| | DSQ02 | 0.552 | 0.489 | | | 0.612 | |
| | DSQ03 | 0.592 | 0.634 | | | | |
| | DSQ04 | 0.585 | 0.541 | | | 0.539 | |
| | DSQ05 | 0.536 | 0.472 | | | 0.606 | |
| | DSQ06 | 0.548 | 0.587 | | | | |
| | DSQ07 | 0.740 | 0.693 | 0.359 | | | |
| | DSQ08 | 0.633 | 0.678 | | | | |
| | DSQ09 | 0.703 | 0.625 | | 0.500 | | |
| | DSQ10 | 0.712 | 0.768 | | | | |
| | DSQ11 | 0.730 | 0.715 | 0.259 | | | |
| | DSQ12 | 0.741 | 0.673 | | 0.471 | | |
| | DSQ13 | 0.731 | 0.615 | | 0.663 | | |
| | DSQ14 | 0.516 | 0.553 | | | | |
| | DSQ15 | 0.841 | 0.664 | 0.712 | | | |
| | DSQ16 | 0.826 | 0.656 | 0.572 | | | |
| Omega-t | | | 0.945 | | | | |
| Omega-h | | | 0.865 | | | | |
| Omega-s | | | | 0.297 | 0.371 | 0.471 | |
| ECV | | | 0.686 | 0.109 | 0.096 | 0.109 | |
