# Supplementary material for: Assessing measurement equivalence of the Danish and Dutch Four-Dimensional Symptom Questionnaire using differential item and test functioning analysis
Source: Scand J Public Health. 2020 Jul 27;49(4):479–86. doi: 10.1177/1403494820942074 (PMC8135249; doi:10.1177/1403494820942074)
Supplement: SJP942074_Supplemental_Table_3 – Supplemental material for Assessing measurement equivalence of the Danish and Dutch Four-Dimensional Symptom Questionnaire using differential item and test functioning analysis [file SJP942074_Supplemental_Table_3.pdf]

Supplementary Table 3. Dimensionality statistics of the bifactor models, by 4DSQ scale and language group

Scale/Age group		PUC	ECV	Omega-h
Distress				
	Danish	0.858	0.706	0.880
	Dutch	0.808	0.787	0.922
Depression				
	Danish	0.733	0.828	0.887
	Dutch	0.733	0.912	0.944
Anxiety				
	Danish	0.848	0.883	0.906
	Dutch	0.909	0.931	0.941
Somatization				
	Danish	0.867	0.628	0.841
	Dutch	0.900	0.686	0.865

PUC = proportion of uncontaminated correlations; ECV = explained common variance; Omega-h = omega-hierarchical
