# Supplementary material for: Assessing measurement equivalence of the Danish and Dutch Four-Dimensional Symptom Questionnaire using differential item and test functioning analysis
Source: Scand J Public Health. 2020 Jul 27;49(4):479–86. doi: 10.1177/1403494820942074 (PMC8135249; doi:10.1177/1403494820942074)
Supplement: SJP942074_Supplemental_Table_4 – Supplemental material for Assessing measurement equivalence of the Danish and Dutch Four-Dimensional Symptom Questionnaire using differential item and test functioning analysis [file SJP942074_Supplemental_Table_4.pdf]

Supplementary Table 4. Item parameters by language group (items with DIF highlighted)

Scale/Items	Danish			Dutch		
**Distress**	***a***	***b1***	***b2***	***a***	***b1***	***b2***
#17	3.088	-0.685	0.063	3.088	-0.685	0.063
#19	2.786	-1.377	-0.469	2.786	-1.377	-0.469
#20	1.603	-1.538	-0.462	1.603	-1.538	-0.462
#22	3.080	-1.527	-0.530	2.630	-0.702	0.024
#25	2.324	-1.047	-0.042	2.890	-1.177	-0.369
#26	2.068	-1.033	-0.030	2.068	-1.033	-0.030
#29	4.289	-0.056	0.482	3.414	-0.350	0.301
#31	3.319	-0.119	0.614	3.319	-0.119	0.614
#32	5.636	0.067	0.643	3.867	-0.293	0.363
#36	4.760	0.060	0.623	4.306	-0.198	0.410
#37	4.034	-0.291	0.373	4.034	-0.291	0.373
#38	2.657	-0.631	0.254	2.657	-0.631	0.254
#39	1.355	-1.038	0.048	1.355	-1.038	0.048
#41	1.970	-1.025	-0.065	1.970	-1.025	-0.065
#47	1.493	-0.501	0.567	1.493	-0.501	0.567
#48	2.106	-0.409	0.451	1.540	-0.229	0.601
**Depression**						
#28	4.002	-0.135	0.488	4.002	-0.135	0.488
#30	6.689	0.401	0.872	6.689	0.401	0.872
#33	7.864	0.618	1.104	7.864	0.618	1.104
#34	3.057	-0.173	0.578	3.057	-0.173	0.578
#35	3.223	-0.029	0.587	3.223	-0.029	0.587
#46	6.143	0.691	1.228	6.143	0.691	1.228
**Anxiety**						
#18	4.714	0.173	0.778	2.167	0.168	0.872
#21	3.413	-0.222	0.479	3.413	-0.222	0.479
#23	2.109	0.574	1.315	2.109	0.574	1.315
#24	3.552	0.354	0.974	3.552	0.354	0.974
#27	3.618	0.445	1.108	4.051	-0.094	0.562
#40	2.767	0.837	1.328	2.767	0.837	1.328
#42	2.493	0.764	1.386	2.502	0.499	1.046
#43	2.632	1.009	1.480	2.632	1.009	1.480
#44	2.029	0.491	1.220	2.029	0.491	1.220
#45	2.469	0.955	1.525	2.469	0.955	1.525
#49	2.518	0.690	1.300	2.573	1.012	1.475
#50	1.717	1.370	2.211	1.436	0.880	1.707
**Somatization**						
#01	1.559	0.165	1.399	1.732	-0.614	0.559
#02	1.610	-0.203	0.675	1.019	-1.208	-0.220
#03	1.456	2.132	3.062	1.456	2.132	3.062
#04	1.255	-0.780	0.171	1.255	-0.780	0.171
#05	1.403	-0.757	0.352	0.967	-1.105	0.074
#06	1.466	-0.249	0.787	1.157	-0.418	0.556
#07	1.785	-0.637	0.580	1.953	-0.069	0.864
#08	1.466	-1.189	0.087	1.466	-1.189	0.087
#09	1.902	-0.671	0.143	1.542	-0.276	0.592
#10	1.896	-0.162	0.794	1.896	-0.162	0.794
#11	2.003	0.048	0.912	2.003	0.048	0.912

#12	2.148	-0.650	0.323	1.741	-0.304	0.599
#13	2.166	-0.486	0.535	1.540	-0.069	0.776
#14	1.424	0.357	1.540	1.044	0.202	1.232
#15	2.106	-0.128	0.816	2.106	-0.128	0.816
#16	2.099	0.391	1.267	2.099	0.391	1.267

a: discrimination parameter

b1 and *b2*: severity parameters
